# Submental tracheal intubation in oromaxillofacial surgery

**DOI:** 10.4103/0970-0358.41105

**Published:** 2008

**Authors:** Ramesh Kumar Sharma, Puneet Tuli, Chacko Cyriac, Atul Parashar, Surinder Makkar

**Affiliations:** Department of Plastic Surgery, Postgraduate Institute of Medical Education and Research Chandigarh, India

**Keywords:** Avoiding tracheostomy, oromaxillofacial surgery, intubation

## Abstract

**Background:**

Oromaxillofacial surgical procedures present a unique set of problems both for the surgeon and for the anesthesist. Achieving dental occlusion is one of the fundamental aims of most oromaxillofacial procedures. Oral intubation precludes this surgical prerequisite of checking dental occlusion. Having the tube in the field of surgery is often disturbing for the surgeon too, especially in the patient for whom skull base surgery is planned. Nasotracheal intubation is usually contraindicated in the presence of nasal bone fractures seen either in isolation or as a component of Le Fort fractures. We utilized submental endotracheal intubation in such situations and the experience has been very satisfying.

**Materials and Methods:**

The technique has been used in 20 patients with maxillofacial injuries and those requiring Le Fort I approach with or without maxillary swing for skull base tumors. Initial oral intubation is done with a flexo-metallic tube. A small 1.5 cm incision is given in the submental region and a blunt tunnel is created in the floor of the mouth staying close to the lingual surface of mandible and a small opening is made in the mucosa. The tracheal end of tube is stabilized with Magil′s forceps, and the proximal end is brought out through submental incision by using a blunt hemostat taking care not to injure the pilot balloon. At the end of procedure extubation is done through submental location only.

**Results:**

The technique of submental intubation was used in a series of twenty patients from January 2005 to date. There were fifteen male patients and five female patients with a mean age of twenty seven years (range 10 to 52). Seven patients had Le Fort I osteotomy as part of the approach for skull base surgery. Twelve patients had midfacial fractures at the Le Fort II level, of which 8 patients in addition had naso-ethomoidal fractures and 10 patients an associated fracture mandible. Twelve patients were extubated in the theatre. Eight patients had delayed extubation in the post-operative ward between 1 and 3 days postoperatively.

**Conclusion:**

In conclusion, the submental intubation technique has proved to be a simple solution for many a difficult problem one would encounter during oromaxillofacial surgical procedures. It provides a safe and reliable route for the endotracheal tube during intubation while staying clear of the surgical field and permitting the checking of the dental occlusion, all without causing any significant morbidity for the patient. Its usefulness both in the emergency setting and for elective procedures has been proved. The simplicity of the technique with no specialized equipment or technical expertise required makes it especially advantageous. This technique therefore, when used in appropriate cases, allows both the surgeon and the anesthetist deliver a better quality of patient care.

## INTRODUCTION

In patients planned for oro-maxillary surgery with limited airway options, the submental intubation has been suggested as an alternative technique. This technique offers a secure airway to the anesthetist, an optimal operating field and an opportunity to check the dental occlusion for the surgeon with limited morbidity for the patient. Since the first description of this technique,[1] several modifications have been suggested. [2–8] We describe here our experience with this technique.

## MATERIALS AND METHODS

Initially oral endotracheal intubation is performed with a 32G flexometallic endotracheal tube (Rusch, Germany). After packing the throat, the connector is checked for proper fit into the tube so that it can be easily removed and reattached in the next step. Under sterile conditions, a 1.5 cm skin crease incision is made in the submental region just medial to the lower border of the mandible, one third of the way from the symphysis to the angle of the mandible. When being used for faciomaxillary injuries, the side used for the incision is dictated by the presence of a concurrent mandible fracture, staying as far away from the fracture site as possible in order to reduce the incidence of interference from the tube. Using a mouth gag the mouth opening is maintained. The floor of the mouth is exposed by retracting the tongue. A closed artery forceps is introduced through the submental skin incision and using blunt dissection a tunnel is created close to the lingual aspect of the mandible in order to avoid injury to the submandibular duct and the lingual nerve located medial to the incision. The endotracheal tube is then disconnected from the breathing circuit and the connector removed. The pilot balloon is then grasped with an artery forceps and pulled out gently. While stabilizing the tracheal end of the tube with a Magill′s forceps, the proximal end of the tube is pulled out through the tunnel created using gentle rotational movement. The connector and breathing system are reattached. The tracheal tube now lies in the floor of the mouth between the tongue and the mandible. After ensuring bilaterally equal air entry, the endotracheal tube position is secured with skin sutures. Free ended sutures are also placed for closure of the incision wound if the patient is to be extubated from the submental region.

The midline technique involves the use of a transverse midline incision premarked in the submental crease at the same level as described above. Staying in the midline, blunt dissection is performed between the anterior bellies of the digastric, mylohyoid, geniohyoid and genioglossus muscles. Intraorally, a longitudinal incision is made in the midline between the submandibular ducts close to the base of the tongue. The tunnel is made wide enough to accommodate the endotracheal tube.

At the end of the procedure, extubation is done through the external skin incision. No attempt is made to close the intraoral incision. The skin incision is however closed using the sutures placed at intubation. In other situations, when opening the intraoperative intermaxillary fixation is permissible, the endotracheal tube is withdrawn into the oral cavity before the patient is extubated. This is also done if a prolonged period of intubation is required.

**Figure 1:**
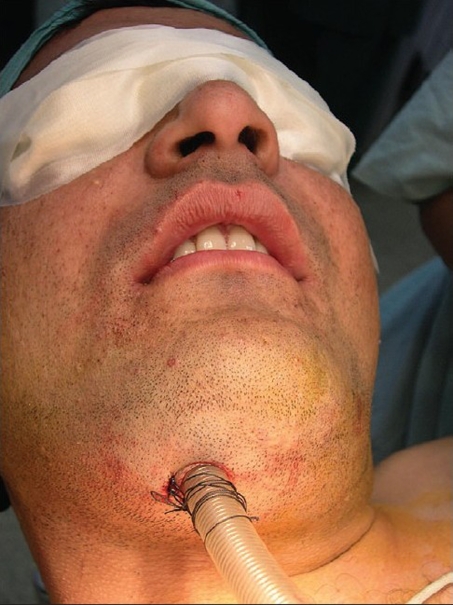
Submental intubation in place in a patient with clivial chordoma

**Figure 2:**
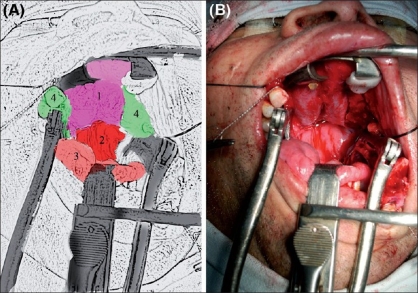
Excellent exposure after le fort 1 osteotomy with maxillary swing. There is no obstruction of the field by the endotracheal tube A Key to Figure 2 1 - nasal mucosa; 2 - posterior pharyngeal wall; 3 - tongue; 4 - maxillary halves that have been swung open

**Figure 3:**
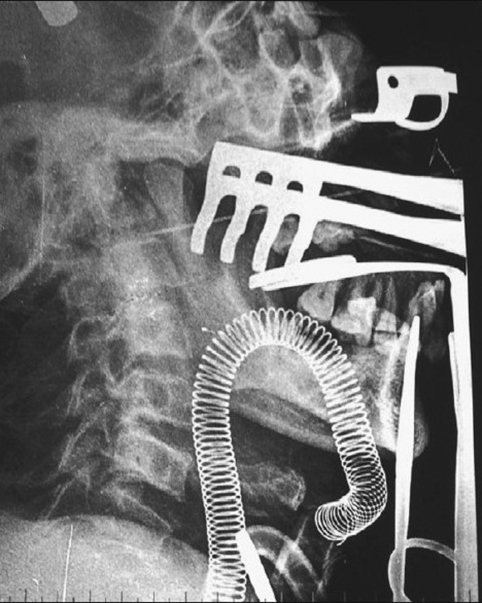
Intraoperative radiograph showing course of the submental intubation

Figure 1 shows a patient of clivial chordoma necessitating a le fort 1 osteotomy with maxillary swing. The submental intubation is in place. [Figure 2] demonstrates excellent exposure allowed because of unobtrusive location of the endotracheal tube. An intraoperative radiograph shows the course of the endotracheal tube through the sub mental route [Figure 3]

## RESULTS

The technique of submental intubation was used in a series of twenty patients from January 2005 to date. There were fifteen male patients and five female patients with a mean age of twenty seven years (range 10 to 52). Seven patients had Le Fort I osteotomy as part of the approach for skull base surgery. Twelve patients had midfacial fractures at the Le Fort II level, of which 8 patients in addition had naso-ethomoidal fractures and 10 patients an associated fracture mandible. All twelve patients had evidence of CSF leak through the anterior nares. One patient was being operated for a post-traumatic class III malocclusion [Table 1].

**Table 1:** Showing details of the patients

*Age/sex*	*Indication for surgery*	*Surgical procedure done*	*Intubation technique*	*Complications*
21	B/L LF II + # mandible	ORIF	SM - L	Nil
37	B/L LF II + # NEC + # mandible	ORIF	SM - L	Nil
42	Clival Chordoma	LF I osteotomy + maxillary swing	SM - M	Nil
45	Basal invagination	LF I osteotomy + maxillary swing	SM - M	Nil
25	B/L LF II + # NEC	ORIF	SM - M	Nil
30	Clival Chordoma	LF I osteotomy + maxillary swing	SM - M	Nil
33	B/L LF II + # mandible	ORIF	SM - L	Nil
27	B/L LF II + # NEC + # mandible	ORIF	SM - L	Nil
19	B/L LF II + # NEC + # mandible	ORIF	SM - M	Nil
44	Clival Chordoma	LF I osteotomy + maxillary swing	SM - M	Nil
24	Post-traumatic Class III malocclusion	LF I osteotomy	SM - M	Nil
26	B/L LF II + # NEC + # mandible	ORIF	SM - M	Nil
10	Clival Chordoma	LF I osteotomy + maxillary swing	SM - L	Wound infection
25	B/L LF II + # NEC + # mandible	ORIF	SM - M	Nil
	Basal invagination	LF I osteotomy + maxillary swing	SM - M	Nil
18	B/L LF II + # mandible	ORIF	SM - M	Nil
20	B/L LF II + # NEC + # mandible	ORIF	SM - L	Nil
23	B/L LF II + # NEC + # mandible	ORIF	SM - M	Nil
52	Pituitary tumor	LF I osteotomy + maxillary swing	SM - M	Nil
19	B/L LF II + # mandible	ORIF	SM - L	Partial extubation

SM - submental intubation, M - midline approach, L - lateral approach, B/L - bilateral, NEC - nasoethmoid complex, ORIF - open reduction internal fixation, LF - leFort

Thirteen patients underwent submental intubation through the midline approach, while the lateral approach was used for the remaining seven. The average time taken to perform this procedure was nine minutes (6-15 minutes). Twelve patients were extubated in the theatre. Eight patients had delayed extubation in the post-operative ward between 1 and 3 days postoperatively.

**Figure 4:**
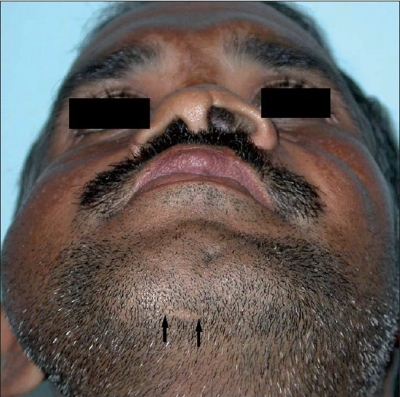
Healed scar of at 6 months (between arrows). The scar is hardly perceptible

Minor complications were encountered in two patients during the submental tracheal intubation procedure. In one pediatric patient, an accidental partial extubation occurred when the tube was being pulled through the submental incision. Wound infection developed in the skin incision site in one patient which settled with regular dressings and antibiotics. All patients were satisfied with the aesthetic appearance of the scar in the submental region irrespective of the approach used [Figure 4].

## DISCUSSION

Oromaxillofacial surgical procedures present a unique set of problems both for the surgeon and for the anesthesist. Achieving dental occlusion is one of the fundamental aims of most oromaxillofacial procedures. Oral intubation precludes this surgical prerequisite of checking dental occlusion. Doing an oral intubation and then positioning the tube in the retromolar space or in the gaps formed due to the loss of teeth would permit this. But the space available is often inadequate to permit the passage of the bulky flexo-metallic endotracheal tube. Having the tube in the field of surgery is often disturbing for the surgeon too, especially in the patient for whom skull base surgery is planned.

Nasotracheal intubation is usually contraindicated in the presence of nasal bone fractures seen either in isolation or as a component of LeFort fractures.[9,10] Panfacial fractures might be associated with CSF leak and nasotracheal intubation in these patients is wrought with danger if it results in the inadvertent introduction of the tube into the cranium.[11] Besides, the deformed anatomical passages might obstruct the endotracheal tube or cause hemorrhage or the displacement of bony fragments.[10,12–14] Even using a fiber-optic bronchoscope in such situations will not entirely abolish the associated dangers.

Conventional tracheostomy, as an alternative, has many inherent complications like hemorrhage, subcutaneous emphysema, pneumomediastinum, pneumothorax, recurrent laryngeal nerve palsy, tracheomalacia, tracheal stenosis, tracheal erosions, stomal and respiratory tract infections, problems with decanulation and cosmetic disfigurement.[15–17] The significant morbidity that can result after tracheostomy necessitates that it should not be used indiscriminately.

The technique of submental intubation is a solution for most of these problems. The advantages of this technique are manifold and it is appreciated by all members of the team. To the anesthetist it offers a secure airway, to the surgeon, an optimal operating field and the opportunity to check the dental occlusion and to the patient, minimal morbidity.

Ever since it was first described by Altemir way back in 1986,[1] the technique of submental intubation has undergone various modifications and found new indications. [2–8] Procedures where submental intubation is indicated and can be used safely would include patients with midface or panfacial fractures with possible base of skull fractures, patients undergoing elective LeFort osteotomies, patients undergoing simultaneous elective mandibular orthognathic surgery and rhinoplasty procedures[9,14,18] [Table 1].

Contraindications for this technique are infection at the site of incision, bleeding diathesis, disrupted larygotracheal anatomy and a restricted retromolar space to allow suctioning.[19] Submental intubation is generally contraindicated where more long-term control of the airway is required or when a more permanent airway is required, i.e, gun shot injuries.[9] Inability to open the mouth was considered a contraindication but Arya *et al.*, have used the pharyngeal loop assembly and described the technique of retrograde submental intubation in order to avoid a short term tracheostomy.[19]

Paetkau *et al.*, locates the skin incision midway between the angle of the mandible and the symphysis and just medial to the lower border of the mandible.[4] But care must be taken to avoid injury to the lingual nerve, submandibular duct and sublingual gland which are located medial to this proposed incision site. Amin *et al.*, suggested locating the incision one-third of the way from the symphysis to the angle of the mandible and emphasized the need to stay close to the lingual surface of the mandible in order to avoid damaging these structures.[14] Excessive bleeding is sometimes seen with the paramedian approach due to injury to the submental vessels. MacInnis described the use of the midline incision in order to avoid this.[3] A midline incision would also ensure that no injury occurs to the lingual nerve, submandibular duct and sublingual gland. The relatively avascular plane in between the two bellies of the mylohyoid and the anterior bellies of the digastric has been suggested as another advantage of the midline technique. The midline incision heals almost imperceptibly and is therefore suggested to be cosmetically superior.[20]

Complications described with the technique of submental intubation would include the displacement of the tube and consequently, accidental extubation.[14] Fixing the tube adequately with sutures would ensure that this does not happen. Accidental partial extubation occurred in only one patient in our series during placement of the submental tube before it was fixed. Amin *et al.* have described the use of capnagraphy during the process of conversion of orotracheal to submental and throughout the surgery to confirm the position of the tube and to serve as a warning tool against accidental extubation.[14] Infection, orocutaneous fistula and postoperative submental scarring have been rarely associated with this technique.[9] Wound infection was observed in only one patient and was managed conservatively. Postoperative submental scarring has been acceptable irrespective of the approach used as it is generally hidden from direct view in the submental region.[9] Paetkau *et al.*, do not suture the submental incision and leave it instead to heal by secondary intention.[4] In our institution, we suture the skin incision alone. The risk of injury to the lingual nerve, submandibular duct or the sublingual gland when using the lateral approach has already been described above. This was however not seen among the patients in our series in whom this approach was used.

In conclusion, the submental intubation technique has proved to be a simple solution for many a difficult problem one would encounter during oromaxillofacial surgical procedures. It provides a safe and reliable route for the endotracheal tube during intubation while staying clear of the surgical field and permitting the checking of the dental occlusion, all without causing any significant morbidity for the patient. Its usefulness both in the emergency setting and for elective procedures has been proved. The simplicity of the technique with no specialized equipment or technical expertise required makes it especially advantageous. This technique therefore, when used in appropriate cases, allows both the surgeon and the anesthetist deliver a better quality of patient care.
